# Whole-Genome Sequencing of Drug-Resistant *Mycobacterium tuberculosis* Strains, Tunisia, 2012–2016

**DOI:** 10.3201/eid2503.181370

**Published:** 2019-03

**Authors:** Imen Bouzouita, Andrea Maurizio Cabibbe, Alberto Trovato, Henda Daroui, Asma Ghariani, Basma Midouni, Leila Essalah, Emna Mehiri, Daniela Maria Cirillo, Leila Slim Saidi

**Affiliations:** National Reference Laboratory for Mycobacteria, Ariana, Tunisia (I. Bouzouita, H. Daroui, A. Ghariani, B. Midouni, L. Essalah, E. Mehiri, L. Slim Saidi);; University of Tunis El Manar, Tunis, Tunisia (I. Bouzouita, B. Midouni)**;**; San Raffaele Scientific Institute, Milan, Italy (A.M. Cabibbe, A. Trovato, D.M. Cirillo);; University of Monastir, Monastir, Tunisia (A. Ghariani, E. Mehiri, L. Slim Saidi)

**Keywords:** WGS, MDR TB, XDR TB, cgMLST, drug resistance marker, tuberculosis and other mycobacteria, Tunisia, whole-genome sequencing, multidrug-resistant, extensively drug-resistant, antimicrobial resistance, bacteria, Mycobacterium tuberculosis

## Abstract

To investigate transmission of drug-resistant strains of *Mycobacterium tuberculosis* in Tunisia, we performed whole-genome sequencing on 46 multidrug-resistant strains isolated during 2012–2016. Core-genome multilocus sequence typing grouped 30 strains (65.2%) into 3 clusters, indicating extensive recent transmission and Haarlem clone predominance. Whole-genome sequencing might help public health services undertake appropriate control actions.

The emergence of drug-resistant strains of *Mycobacterium tuberculosis* is hampering the control of tuberculosis (TB) worldwide. In Tunisia in 2017, the estimated percentage of TB patients with multidrug-resistant (MDR)/rifampin-resistant TB was 1.1% among those with new infection and 13% among those with previously treated infection (*1*).

Effective and rapid tools are needed to characterize and track the transmission chains of MDR/rifampin-resistant TB. Whole-genome sequencing (WGS) has shown higher discriminatory power for epidemiologic investigations than have other conventional genotyping methods (e.g., spoligotyping, *IS*6110 restriction fragment length polymorphism, and mycobacterial interspersed repetitive unit–variable-number tandem repeat). Indeed, WGS has enabled investigators to rule out false transmission events (*2*–*7*). Furthermore, WGS enables simultaneous determination of polymorphisms and insertions/deletions linked to resistance to first-line and second-line drugs (*8*).

In this study, we used WGS to investigate transmission of MDR and extensively drug resistant (XDR) TB strains isolated in Tunisia over a 4-year period by applying the core-genome multilocus sequence typing (cgMLST) scheme and identifying the drug-resistance marker for first-line and second-line drug resistance. This study was approved by the ethics committee of A. Mami Pneumology Hospital, Ariana, Tunisia.

## The Study

We retrospectively studied 46 MDR *M. tuberculosis* isolates collected from 46 HIV-negative patients in Tunisia during June 2012–June 2016, which represented 57 (80.7%) cases of MDR TB. Of the 46 isolates, 6 represented all (100%) XDR TB cases recorded in the country during that period. We performed drug-susceptibility testing for resistance to first-line drugs (except pyrazinamide) by using the proportion method on Lowenstein-Jensen medium. For pyrazinamide and second-line drugs, we performed drug-susceptibility testing on a Bactec MGIT 960 system (Becton, Dickinson and Company, http://www.bd.com). WGS was performed on the MiniSeq platform (Illumina Inc., https://www.illumina.com) targeting a minimum average reads coverage of 50-fold. To analyze the mutations involved in drug resistance and related to lineage determination, we used PhyResSe and TGS TB (*9*,*10*). We performed the cgMLST scheme version 2.1, considering 2,891 core genes, by using Ridom SeqSphere+ version 5.0.0 software (Ridom GmbH, https://www.cgmlst.org) (*7*). To define a strain as a part of a recent transmission chain, we fixed a threshold of <6 allele variants. For statistical analyses, we calculated p values by using OpenEpi version 3 (https://www.openepi.com) and considered p<0.05 to be significant.

**Figure 1:**
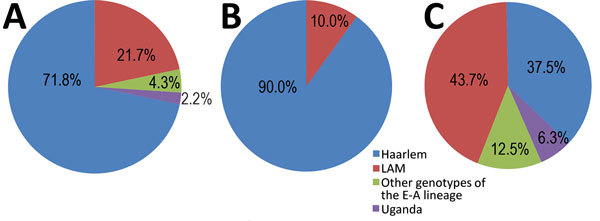
Lineage and family distribution of drug-resistant strains identified during study of drug-resistant *Mycobacterium tuberculosis*, Tunisia, 2012–2016. A) MDR strains. Haarlem was the most frequently represented family (71.8%) among MDR strains. Among other samples, 21.7% belonged to the LAM family, 4.3% presented other genotypes of the EA lineage, and 2.2% the Uganda genotype. B) Clustered MDR strains. Haarlem was also the most frequent family (90.0%); the rest belonged to the LAM family. C) Nonclustered MDR strains. Among nonclustered isolates, LAM was the most frequently represented family (43.7%), followed by Haarlem, which was detected in 37.5% of the strains. EA, European-American lineage; LAM, Latin-American-Mediterranean lineage; MDR, multidrug-resistant.

Patient origins are reported in the Appendix. Most MDR TB cases (19; 41.3%) were recorded in the Bizerte region in northern Tunisia. WGS revealed that all MDR/XDR strains belonged to the European-American lineage (lineage 4); the Haarlem family was the most frequent (71.8%) (Figure 1). (WGS files from this study have been submitted to the European Nucleotide Archive as fastq files under study accession no. PRJEB30463, https://www.ebi.ac.uk/ena/data/search?query=PRJEB30463).

**Figure 2:**
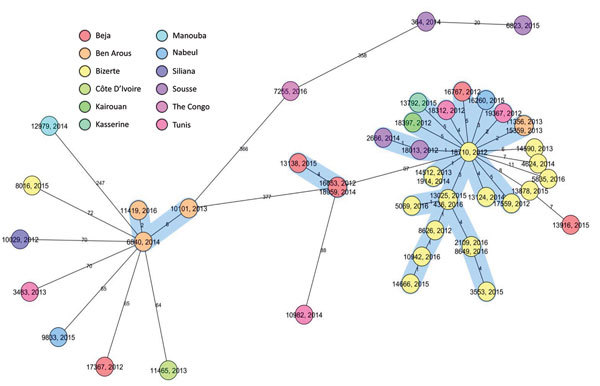
Core-genome multilocus sequence typing–based minimum spanning tree for strains identified during study of drug-resistant *Mycobacterium tuberculosis*, Tunisia, 2012–2016. Ridom SeqSphere+ minimum spanning tree for 46 samples based on 2,891 columns, pairwise ignoring missing values, logarithmic scale. Cluster distance threshold = 5 alleles. Colors indicate regions and countries of patient origin.

The cluster analysis (Figure 2) showed that 30 (65.2%) of 46 isolates were grouped within 3 clusters, and most (90.0%) clustered MDR TB cases belonged to the Haarlem family (Figure 1). Of note, a big cluster of 24 MDR strains linked to Haarlem (cluster 1) was detected over the entire study period. We found no significant associations between this cluster and patient sex, age (<35 years, >35 years), and resistance to second-line drugs (XDR TB) (p>0.05); however, we found a significant association with this cluster and pyrazinamide resistance (p = 0.001) and with Bizerte (p = 0.014). Despite the association with the Bizerte region, patients from various regions were part of this cluster (Figure 2). We also found a significant association between cluster 2 (Haarlem) and Beja (p<0.001) and between cluster 3 (Latin-American-Mediterranean) and Ben Arous (p**<**0.001). Epidemiologic links were confirmed for patients in these 2 transmission chains (Table 1).

**Table 1:** Epidemiologic information about clustered cases identified during study of drug-resistant *Mycobacterium tuberculosis* strains, Tunisia, 2012–2016*

Cluster, group	***pncA* mutation**	**No. patients**	**Epidemiologic links**
Cluster 1, Haarlem†			
Group 1	Gln10His	9	Confirmed for patients 10942, 14666 (2 brothers with XDR TB), 8626 (their neighbor with XDR TB) in Bizerte, and patients 13025 and 2109 (2 friends from Bizerte); probable for patients 3553, 436, 5069, 8649 (from Bizerte)
Group 2	Leu4Trp	5	Probable for patients 18013 and 2666 (from Sousse, the central part of Tunisia)
Group 3	A-11C	4	Not available
Group 4	CA deletion (+339, +340)	2	Probable for patients 11356 and 15359 (from Ben Arous, 10 km from the capital Tunis)
Group 5	Cys14Stop	2	Confirmed for patients 1914 and 14512 (brother and sister from Bizerte)
Group 6	Wild type	2	Not available
Cluster 2, Haarlem	Wild type	3	Confirmed for patients 13138, 18959, 16653 (same family: father, son, and uncle; Beja; Northwest of Tunisia)
Cluster 3, LAM	Wild type	3	Confirmed for patients 10101, 11419, 6840 (neighbors from Ben Arous)

*LAM, Latin-American-Mediterranean. †For the remaining patients of cluster 1, no information was available.

All cluster 1 strains shared mutations in drug-resistance genes (e.g., *rpoB*, Ser450Leu, the compensatory mutation *rpoB*Val534Met (*11*), *katG* Ser315Thr, *embB* Met306Ile, and *gidB*Arg47Trp). However, diversity was noticed in *pncA* mutations conferring resistance to pyrazinamide. On the basis of *pncA* variation, this cluster was split into 6 groups (Table 1).

Four XDR isolates belonged to cluster 1. Three XDR isolates from 2 brothers (patients 10942 and 14666) and their neighbor (patient 8626) shared mutations conferring resistance to second-line drugs and to pyrazinamide (Tables 1, 2), supporting evidence of direct transmission of this XDR strain. The fourth XDR TB case (patient/strain 13792) differed in mutations conferring resistance to pyrazinamide and fluoroquinolones. This case probably shared an ancestral MDR strain with cluster 1, which had evolved differently because of poor adherence of the patient to drug therapy (Table 2; Appendix). Five nonclustered MDR isolates linked to Haarlem (4 patients from Bizerte) were distant by 6–11 alleles from cluster 1 showing the same polymorphisms in *rpoB, katG, embB*, and *gidB* (Figure 2; Appendix). Of note, cluster 2 presented the compensatory mutation *rpoB* Val534Met, but no variation in *embB*, *gidB*, and *pncA* was detected (Appendix). This Haarlem cluster showed limited transmission compared with cluster 1, which was distant by 97 alleles (Figure 2).

**Table 2:** Mutations detected in genes conferring resistance to fluoroquinolones, aminoglycosides, and capreomycin in study of drug-resistant *Mycobacterium tuberculosis* strains, Tunisia, 2012–2016*

No. strains	**Cluster**	**Resistance to FQ, AG, or CAP on MGIT 960**	***gyrA***	***gyrB***	***rrs***	***tlyA***	***eis*–p**
3	1	OFX, LVX, KAN, AMK, CAP	Asp94Gly†		A1401G†		
1	1	OFX, LVX, KAN, AMK, CAP		Asp461His/Gly470Cys	A1401G†		
1	1	OFX, LVX	Ala90Val†				
1	1	OFX, LVX	Asp94Gly†				
1	3	OFX, LVX	Ser91Pro†				
1	Not clustered	OFX, LVX, KAN	Asp94Ala†	Asp461Asn			C-14T†
1	Not clustered	OFX, LVX, CAP	Ser91Pro†			INS of 2G†	
1	Not clustered	OFX, LVX	Asp94Ser				
1	Not clustered	OFX, LVX	Ala90Val†				
1	Not clustered	OFX, LVX		Asp461Asn			
1	Not clustered	OFX, LVX	Asp94Tyr†				

*Among second-line drugs, 7 strains were pre-XDR (FQ resistant) and 6 were XDR TB strains on MGIT 960. AG, aminoglycosides; AMK, amikacin; CAP, capreomycin; eis-p, eis promoter; FQ, fluoroquinolone; INS, insertion; KAN, kanamycin; LVX, levofloxacin, MGIT 960, Bactec MGIT 960 system (Becton, Dickinson and Company, http://www.bd.com); OFX, ofloxacin; TB, tuberculosis; XDR, extensively drug resistant.

†High-confidence mutations associated with FQ, AG, or CAP resistance according to (*12*).

In our study, mutation *rpoB*Ser450Leu was mostly associated with rifampin resistance (n = 42, 91.3%), whereas codon 315 of *katG* was most involved with isoniazid resistance (n = 43, 93.4%). Three nonclustered MDR isolates presented a genomic deletion of ≈10.4 kb, which included the entire *katG* gene (strain 3483) and 2 uncommon mutations in *katG*: Gly269Asp and Gly279Asp (patients/strains 9833, 10982) (Appendix). All pyrazinamide-resistant strains detected with the MGIT 960 system (n = 33, 71.7%) had a mutation in the *pncA* gene or its promoter.

Regarding second-line drugs, all 13 fluoroquinolone-resistant strains had a mutation in the quinolone resistance–determining region of *gyrA*, *gyrB*, or both*.* For second-line injectable drugs, 4 XDR strains had the mutation *rrs* A1401G and 2 had the mutation *eis* C-14T and a frame shift in *tlyA* (Table 2).

## Conclusions

cgMLST analysis showed that 65.2% of MDR/XDR strains of *M. tuberculosis* were clustered, reflecting extensive transmission in Tunisia, particularly of a Haarlem clone. This Haarlem clone showed polymorphisms *rpoB* Ser450Leu, Val534Met, *katG* Ser315Thr, *embB* Met306Ile, and *gidB*Arg47Trp, and in *pncA* genes previously identified in a Haarlem MDR TB outbreak in the Bizerte region during 2001–2011 (*13*,*14*). As indicated by statistical association, we conclude that this cluster is still spreading in the Bizerte area. However, diffusion in different regions of the country is alarming and requires intensified efforts to control and diagnose drug-resistant TB.

Only 2 strains, belonging to cluster 1, did not have any mutations in *pncA.* The wild-type *pncA* isolates might represent the genotype of the first strains that emerged in Bizerte and evolved since 2001 by acquiring single-nucleotide polymorphisms in *pncA* and the other genes, including genes involved in resistance to second-line drugs. It has been reported that the mutation rate during a transmission chain or TB latency is not completely stable and is estimated at 0.3–0.5 single-nucleotide polymorphisms/genome/year (*4*,*5*,*15*), which leads to increased numbers of allele variants for some isolates and might explain the results found for 5 MDR strains distant from cluster 1 by 6–11 alleles.

The main limitation of this study is the incomplete number of MDR TB cases (≈19% missing). Epidemiologic information to confirm all clustered cases is lacking.

In summary, cgMLST-based WGS showed extensive transmission of MDR/XDR TB in Tunisia over 4 recent years, thereby indicating that MDR TB is not fully controlled. Use of this molecular approach for surveillance purposes might enable the public health service to undertake appropriate control actions, particularly in specific settings of this country.

Appendix Whole-genome sequencing of 46 multidrug-resistant/extensively drug-resistant strains of *Mycobacterium tuberculosis* isolated in Tunisia, 2012–2016.
